# Noninvasive Visualization of the Activated αvβ3 Integrin in Cancer Patients by Positron Emission Tomography and [^18^F]Galacto-RGD

**DOI:** 10.1371/journal.pmed.0020070

**Published:** 2005-03-29

**Authors:** Roland Haubner, Wolfgang A Weber, Ambros J Beer, Eugenija Vabuliene, Daniel Reim, Mario Sarbia, Karl-Friedrich Becker, Michael Goebel, Rüdiger Hein, Hans-Jürgen Wester, Horst Kessler, Markus Schwaiger

**Affiliations:** **1**Nuklearmedizinische Klinik und PoliklinikTechnische Universität MünchenGermany; **2**Institut für RöntgendiagnostikTechnische Universität MünchenGermany; **3**Institut für PathologieTechnische Universität MünchenGermany; **4**Klinik für Orthopädie und SportorthopädieTechnische Universität MünchenGermany; **5**Klinik und Poliklinik für Dermatologie und Allergologie, Technische UniversitätMünchenGermany; **6**Department Chemie, Lehrstuhl II für Organische ChemieTechnische Universität München, GarchingGermany; Institute of Nuclear MedicineUnited Kingdom

## Abstract

**Background:**

The integrin αvβ3 plays an important role in angiogenesis and tumor cell metastasis, and is currently being evaluated as a target for new therapeutic approaches. Several techniques are being studied to enable noninvasive determination of αvβ3 expression. We developed [^18^F]Galacto-RGD, a ^18^F-labeled glycosylated αvβ3 antagonist, allowing monitoring of αvβ3 expression with positron emission tomography (PET).

**Methods and Findings:**

Here we show by quantitative analysis of images resulting from a small-animal PET scanner that uptake of [^18^F]Galacto-RGD in the tumor correlates with αvβ3 expression subsequently determined by Western blot analyses. Moreover, using the A431 human squamous cell carcinoma model we demonstrate that this approach is sensitive enough to visualize αvβ3 expression resulting exclusively from the tumor vasculature. Most important, this study shows, that [^18^F]Galacto-RGD with PET enables noninvasive quantitative assessment of the αvβ3 expression pattern on tumor and endothelial cells in patients with malignant tumors.

**Conclusions:**

Molecular imaging with [^18^F]Galacto-RGD and PET can provide important information for planning and monitoring anti-angiogenic therapies targeting the αvβ3 integrins and can reveal the involvement and role of this integrin in metastatic and angiogenic processes in various diseases.

## Abstract 2

Visualising integrins with PET scanning can show angiogenesis in tumors and also be used to monitor anti-angiogenic therapy

## Introduction

Cell–cell and cell–matrix interactions play essential roles in tumor metastasis and angiogenesis. Integrins are one of the main classes of receptors involved in these processes. In addition to having adhesive functions, integrins transduce messages via various signaling pathways and influence proliferation and apoptosis of tumor cells, as well as of activated endothelial cells. One prominent member of this receptor class is the integrin αvβ3. It has been demonstrated that αvβ3 is an important receptor affecting tumor growth, local invasiveness, and metastatic potential [1,2]. This integrin is expressed on various malignant tumors and mediates adhesion of tumor cells on a variety of extracellular matrix proteins, allowing these cells to migrate during invasion and extravasation [3].

The integrin αvβ3 is also highly expressed on activated endothelial cells during angiogenesis [4]. In contrast, expression of αvβ3 is weak in resting endothelial cells and most normal organ systems [5]. On activated endothelial cells, the receptor mediates migration through the basement membrane during formation of the new vessel, which is essential for sufficient nutrient supply of the growing tumor. Inhibition of the αvβ3-mediated cell–matrix interaction has been found to induce apoptosis of activated endothelial cells. Thus, the use of αvβ3 antagonists is currently being evaluated as a strategy for tumor-specific anti-cancer therapies [6,7,8]. Owing to the weak expression on non-activated endothelial cells, treatment with αvβ3 antagonists does not affect preexisting blood vessels. Inhibition of blood vessel formation in tumor models using αvβ3 antagonists not only blocks tumor-associated angiogenesis, but in some cases results in tumor regression [9].

However, αvβ3 antagonists can induce apoptosis not only of activated endothelial cells but also of αvβ3-positive tumor cells [10], resulting in a direct cytotoxic effect on tumor cells. Moreover, blocking of the receptor expressed on tumor cells can reduce invasiveness and spread of metastases [11]. Furthermore, αvβ3-binding molecules have been successfully used to “target” a variety of therapeutic agents to the tumor tissue. These include chemotherapeutic agents [12], cDNA-encoding anti-angiogenic genes [13], and T lymphocytes [14].

These encouraging experimental studies have already led to initial clinical trials evaluating the use of αvβ3 antagonists (e.g., vitaxin [15] and cilengitide [16]) in patients with various malignant tumors [17,18,19,20]. Currently available imaging techniques are limited in monitoring treatment with this class of drugs. Anti-tumor activity is generally assessed by determining the percentage of patients in whom a significant reduction in tumor size is achieved during a relatively short period of therapy (“response rate”). Thus, this method may not be applicable for a form of therapy that is aimed at disease stabilization and prevention of metastases. New methods are urgently needed for planning and monitoring treatments targeting the αvβ3 integrin.

Based on cyclo(-Arg-Gly-Asp-DPhe-Val-) [21], a variety of radiolabeled αvβ3 antagonists for single photon emission tomography and positron emission tomography (PET) have been developed (for review see [22,23]). [^18^F]Galacto-RGD (arginine–glycine–aspartic acid), a glycosylated cyclic pentapeptide, resulted from a consequent tracer optimization [24] based on the first-generation peptide [^125^I]-3-iodo-DTyr^4^-cyclo(-Arg-Gly-Asp-DTyr-Val-) [25] and showed high affinity and selectivity for the αvβ3 integrin in vitro, receptor-specific accumulation in αvβ3-positive tumors, and high metabolic stability in a murine tumor model, as well as rapid, predominantly renal elimination [26,27]. Here we describe how [^18^F]Galacto-RGD allows quantification of αvβ3 expression in vivo, show that tumor-induced angiogenesis can be monitored in a murine tumor model, and for the first time, to our knowledge, demonstrate that this class of tracers can be used in patients for noninvasive determination of αvβ3 expression.

## Methods

### Tracer Synthesis

Synthesis of the labeling precursor and subsequent ^18^F-labeling was carried out as described [27]. For application in patients, after high-performance liquid chromatography the collected fraction was evaporated to dryness; 0.5 ml of absolute ethanol and 10 ml of phosphate-buffered saline (pH 7.4) were added; and the product was passed through a Millex GV filter (Millipore, Eschborn, Germany). Quality control of the product was carried out according to the demands of the local regulatory authorities.

### Murine Tumor Models

For in vivo evaluation, xenotransplanted human melanoma models (M21 and M21-L) and a human squamous cell carcinoma model (A431) were used. The M21 cell line expressing αvβ3 [25,28] acted as a positive control and the M21-L cell line, a stable variant cell line of M21 failing to transcribe the αv gene, as a negative control [29]. Cell culture conditions for M21 and M21-L cells are described elsewhere [26]. Similar protocols were used for A431 cells.

The experimental protocol involving animals was approved by the Committee of Veterinarian Medicine of the State of Bavaria; handling of animals was performed according to the standards set by the Committee of Veterinarian Medicine.

In order to study the correlation between αvβ3 expression and tumor uptake of [^18^F]Galacto-RGD, we injected mice subcutaneously with mixtures of M21 and M21-L cells. Pilot experiments had indicated that injection of 1.5 × 10^6^ M21 cells leads within 4 wk to the formation of tumors with a diameter of approximately 8 mm. To obtain similarly sized M21-L tumors, it was necessary to inject 6 × 10^6^ cells. In order to study tumors with approximately 10%, 25%, 50%, and 75% M21 cells, we injected mice with the following mixtures of M21 and M21-L cells: 1.5 × 10^5^ / 5.4 × 10^5^, 3.8 × 10^5^ / 4.6 × 10^6^, 7.5 × 10^5^ / 3 × 10^6^, and 1.1 × 10^6^ / 1.5 × 10^6^. Four weeks after inoculation, nude mice were injected with 7.4 MBq of [^18^F]Galacto-RGD and scanned at the small-animal PET. Subsequently, tumors and other organs of interest were dissected, immediately counted, cut in two pieces, and frozen for further workup.

For experiments with the squamous cell carcinoma model, approximately 10^6^ A431 cells were injected subcutaneously in nude mice. Two weeks after inoculation, 7.4 MBq of [^18^F]Galacto-RGD was injected, and mice were scanned in the animal PET. Animals were sacrificed, and organs of interest were dissected and subsequently weighed and counted or used for immunohistochemical analysis.

### Immunohistochemistry

For immunohistochemical investigation, frozen tumor tissues from mice, as well as from patients, were sectioned (6 μm) and stained using the biotinylated monoclonal anti-αvβ3 antibody LM609 (1:100; Chemicon Europe, Hofheim, Germany). For staining the murine β subunit, a monoclonal hamster anti-mouse antibody (1:10; Chemicon Europe) and a biotinylated mouse anti-hamster IgG secondary antibody (1:200; Chemicon Europe) were used. Sections were processed by peroxidase staining (peroxidase substrate KIT AEC, Vector Laboratories, Burlingame, California, United States).

### Western Blotting

The frozen tumor tissue was homogenized and extracted with lysis buffer (50 mM Hepes (pH 7.5), 150 mM NaCl, 10% Glycerol, 1% Triton X-100, 1 mM EDTA, 1 mM EGTA, 10 mM Na_4_P_2_O_7_, 1 mM MSF, 10 μg/ml Aprotinin, 10 μg/ml Leupeptin). Protein concentration was determined according to Bradford [30] and adjusted to equivalent values using lysis buffer. After SDS-PAGE and transfer, immunoblotting was carried out using a polyclonal rabbit anti-αv antibody (1:500; Chemicon Europe) and a ^125^I-labeled polyclonal donkey anti-rabbit IgG antibody (1:400; 477 kBq/μg, Amersham Buchler, Braunschweig, Germany). For analysis, blots were placed on a phosphor screen for 2 d. For readout out, a Molecular Dynamics PhosphorImager 445 SI (Sunnyvale, California, United States) was used.

### PET Studies with a Small-Animal Scanner

PET imaging of tumor-bearing mice was performed using a prototype small-animal positron tomograph (Munich Avalanche Photodiode PET; [31]). The animal scanner consists of two sectors, comprising three detector modules each, which rotate around the animal for acquisition of complete projections in one transaxial slice (30 angular steps). Each module consists of eight small (3.7 × 3.7 × 12 mm3) lutetium-oxy-orthosilicate crystals read out by arrays of avalanche photodiodes. List mode data are reconstructed using statistical, iterative methods including the spatially dependent line spread function. Reconstructed image resolution is 2.5 mm (full width at half maximum) in a transaxial field of view of 7.5 cm, and the slice thickness is 2 mm. Ninety minutes after the injection of approximately 7.4 MBq of [^18^F]Galacto-RGD, animals were positioned prone inside the tomograph, and a transaxial slice through the tumor region was measured for 480 s

### Patient Study

The study protocol was approved by the ethics committee of the Klinikum Rechts der Isar (Protocol S1), and each patient gave written and informed consent prior to the study (Protocol S2). Nine patients were scanned (five female and four male; age, 26–75 y), who suffered from either malignant melanoma with lymph node metastasis (stage IIIb; patients 1–3), malignant melanoma with distant metastasis (stage IV; patients 4 and 5), chondrosarcoma (patient 6), soft tissue sarcoma (patient 7), osseous metastasis of renal cell carcinoma (patient 8), or villonodular synovitis of the knee (patient 9). Patient selection was focused on melanoma and sarcoma because there is considerable evidence that these tumor types express αvβ3.

Diagnosis prior to scanning was made by biopsy (patients 6–8), by CT (patients 1, 2, and 4–8), by MRI (patient 9), and/or by [^18^F]fluorodeoxyglucose ([^18^F]FDG)–PET (patients 1–4 and 8). After scanning, the diagnosis was confirmed by surgery and histopathological examination of the resectioned specimen (patients 1, 2, 5, 6, 8, and 9) or by combined analysis of morphological imaging, [^18^F]FDG PET, and the patient's clinical data and history (patients 3, 4, and 7).

For immunohistochemistry, sampled specimens (patients 1, 2, 5, 6, 8, and 9) were snap frozen in liquid nitrogen and stored at −70 °C until staining was performed. Tissue samples were taken within 1 wk after scanning from the tumor regions with the maximum tracer uptake. Light microscopic evaluation of the density of αvβ3-positve microvessels was performed as described previously [32]. Briefly, areas with the highest density of αvβ3-positve microvessels were identified using scanning magnification. Subsequently, αvβ3-positve microvessels were counted in three adjacent microscopic fields using a 40× magnifying lens and 10× ocular, corresponding to an area of 0.588 mm^2^. Determination of microvessel density was performed by one senior pathologist (M. S.), who was blinded for the results of the corresponding standardized uptake value (SUV) analysis of tracer accumulation. Then the correlation between the mean values of the vessel counts and the corresponding SUVs was analyzed.

PET scanning was performed using an ECAT Exact PET scanner (Siemens-CTI, Knoxville, Tennessee, United States). After injection of 144–200 MBq of [^18^F]Galacto-RGD, three consecutive emission scans (starting at 7 ± 2.7 min, 37 ± 10.5 min, and 79 ± 18.4 min post injection [p. i.]) from the body stem and, if necessary, from tumor regions outside the body stem were obtained. For one patient, only one scan starting 120 min. p. i. was carried out. Attenuation- and decay-corrected images were reconstructed by using an ordered subsets expectation maximization algorithm. The accumulation of [^18^F]Galacto-RGD was evaluated by calculating the mean SUV normalized to the patient's body weight according to the following formula [33]: (measured activity concentration [Bq/ml] × body weight [kg]) / injected activity [Bq]. The axial slice of the lesion with the maximum activity accumulation was chosen by visual estimation, a region of interest with a diameter of 15 mm was selected on the lesion, and the resulting mean SUV was used for further analysis. For lesions smaller than 2 cm in diameter, a region of interest with a diameter of 10 mm was used and the analysis was based on maximum SUV rather than mean SUV, in order to minimize partial volume effects, which could lead to an underestimation of the SUV.

Dosimetry calculations are based on the MIRDOSE 3.0 program. Data from six patients were analyzed by selecting regions of interest with a diameter of 1.5 cm on the source organs (lung, liver, spleen, kidneys, muscle, bladder, intestine, and heart [left ventricle]). Activity measurements (in Becquerels per cubic centimeter) were performed for all three consecutive scans (mean time p.i. ± standard deviation, 7 ± 2.7 min, 37 ± 10.5 min, and 79 ± 18.4 min, respectively), using a monoexponential fit for calculation of residence times. The volume of the source organs lung, liver, spleen, and kidneys was measured by CT volumetry (Siemens, Forchheim, Germany) in four patients. For the other source organs in these four patients and all organs in the remaining two patients, standardized volume values of the source organs adapted to the patient's body weight were used.

### Statistical Methods

All quantitative data are expressed as mean +/− one standard deviation. The correlation between quantitative parameters was evaluated by linear regression analysis and calculation of Pearson's correlation coefficient. Statistical significance was tested by using analysis of variance (ANOVA).

## Results

### Correlation of Tracer Uptake with αvβ3 Expression

**Figure 1:**
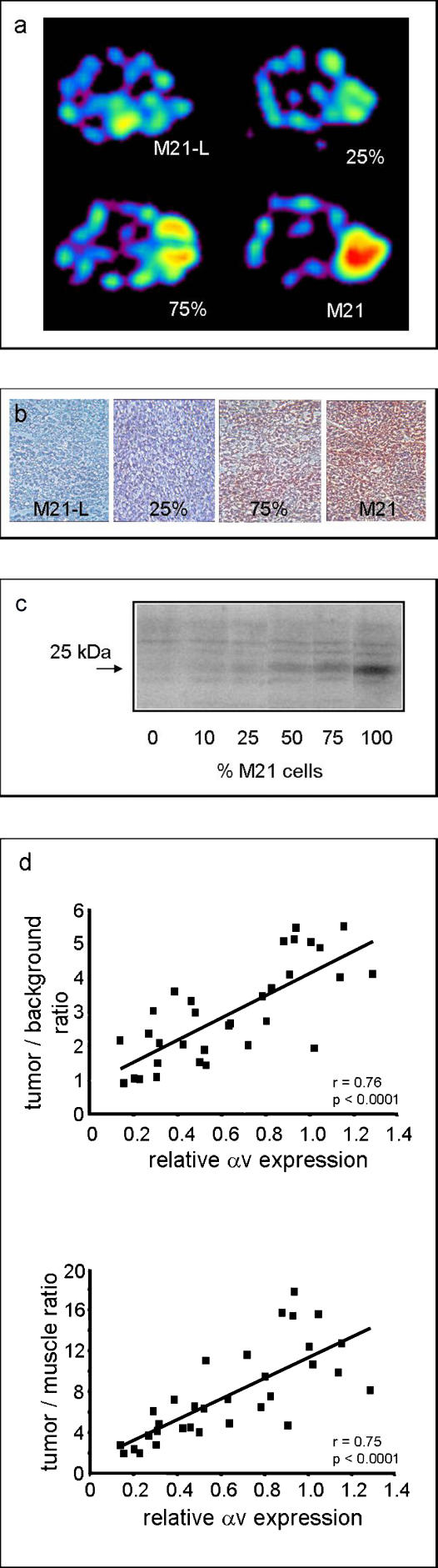
Preclinical Evaluation of [^18^F]Galacto-RGD (A) Transaxial images of nude mice bearing tumors with increasing amounts of αvβ3-positive M21 cells (0% [M21-L], 25%, 75%, and 100% [M21]) 90 min p. i. provided by a prototype small-animal PET scanner show an increasing tracer uptake in the tumor and low background activity. (B) Immunohistochemical staining of tumor tissue sections prepared after PET imaging with an anti-human αvβ3 monoclonal antibody (LM 609) indicate that there is a correlation between tracer uptake and αvβ3 expression. (C) Western blots of the dissected tumors show a band at 25 kDa that corresponds with the mass of the αv subunit under reductive conditions, and indicate the increasing αvβ3 density in the murine tumor model used. (D) The correlation between receptor expression and [^18^F]Galacto-RGD accumulation is confirmed by quantitative analysis based on the tumor/background ratios and tumor/muscle ratios calculated from the PET images and from the biodistribution studies, respectively, and by the relative αv expression in Western blot analyses.

We have previously demonstrated, using a murine tumor model in which the tumor cells are either αvβ3-positive (human melanoma M21) or αv-negative (human melanoma M21-L), that [^18^F]Galacto-RGD shows receptor-specific accumulation in the αvβ3-positive tumor [26]. Here we studied the correlation of [^18^F]Galacto-RGD uptake with the level of αvβ3 expression. We injected tumor cell mixtures containing increasing amounts of αvβ3-positive M21 cells subcutaneously into nude mice. Transaxial images of mice 4 wk after cell inoculation and 90 min after tracer injection using a prototype small-animal PET scanner [31] showed increasing tracer uptake in the tumor corresponding with the percentage of receptor-positive cells (Figure 1A and 1B).

We validated these qualitative results by determining the relative amount of the αv subunit in the dissected tumors through Western blot analysis (Figure 1C). These data were correlated with the tumor/background ratios resulting from the quantitative analysis of the PET images (Figure 1D), as well as with the tumor/muscle ratios resulting from the biodistribution analysis carried out after the PET study (Figure 1D). Both analyses showed a significant correlation between [^18^F]Galacto-RGD and relative αv expression, thus confirming the qualitative analysis by immunohistochemistry.

The systematic difference between tumor/background and tumor/muscle ratios is due to the fact that the region of interest used to define the tumor region in the PET images will always contain not only tumor, but also normal tissues with low [^18^F]Galacto-RGD uptake, such as muscle and lung. This is due to the limited spatial resolution of the PET scanner, which does not allow a sharp distinction between tumor and normal tissue. Accordingly [^18^F]Galacto-RGD uptake by the tumor tissue will be underestimated, and the tumor/background ratio will be lower than the tumor/muscle ratio. Furthermore, tissue sampling was performed 30 min after the start of the PET scan. Clearance of radioactivity from the muscle tissue during this time period will also systematically increase the tumor/muscle ratio compared to the tumor/background ratio calculated from the PET images.

When correlating the weight of the tumor with the relative αv expression, we found a nonsignificant trend for lower αv expression in larger tumors (*r* = 0.34, *p* = 0.09). This is probably related to the presence of necrotic regions in larger tumors, which do not demonstrate αv expression. Thus, it can be excluded that the positive correlation between αv expression and [^18^F]Galacto-RGD uptake is due to systematic differences in the size of tumors.

### Noninvasive Determination of αvβ3 Expression on Endothelial Cells

**Figure 2:**
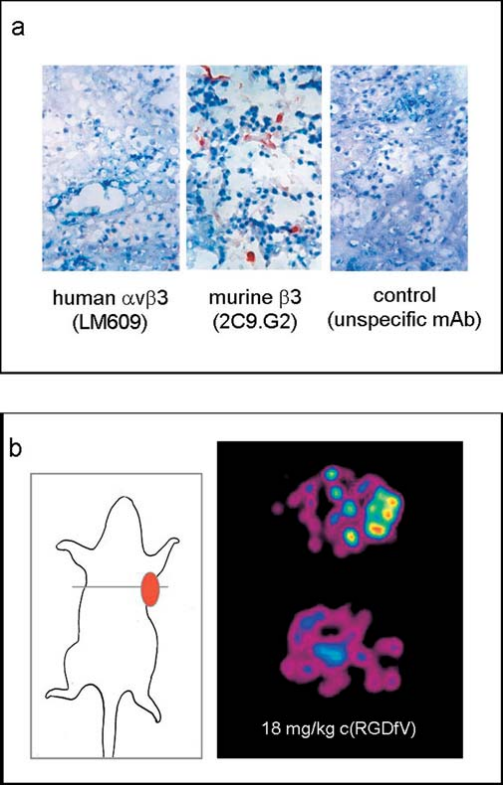
Noninvasive Monitoring of αvβ3 Expression on the Tumor Vasculature (A) Immunohistochemical staining of tumor section using the anti-αvβ3 monoclonal antibody LM609 demonstrates that squamous cell carcinoma cells of human origin do not express the αvβ3 integrin. In contrast, staining of section with an antibody against the murine β3 subunit indicates that the tumor vasculature is αvβ3-positive. (B) Transaxial images of a nude mouse bearing a human squamous cell carcinoma at the right shoulder (left) acquired at the small-animal PET 90 min after tracer injection show a clearly contrasting tumor. Tracer accumulation in the tumor (right, top image) can be blocked by injecting 18 mg of cyclo(-Arg-Gly-Asp-DPhe-Val-) per kilogram of mouse 10 min prior to tracer injection (right, bottom image), indicating receptor-specific accumulation.

To determine whether PET with [^18^F]Galacto-RGD allows noninvasive determination of αvβ3 expression on activated endothelial cells, we used A431 tumor xenografts. A431 cells do not express αvβ3, but induce extensive angiogenesis when subcutaneously transplanted into nude mice. [34]. Immunohistochemical staining of tumor sections using a monoclonal anti-human αvβ3 antibody confirmed that the tumor cells do not express this integrin (Figure 2A). In contrast, staining with a polyclonal antibody against the murine β3 subunit demonstrated expression of β3 on endothelial cells of the tumor vessels. Since αIIbβ3, the only further integrin containing a β3 subunit, is mainly expressed on platelets, it can be excluded that staining depends on this receptor. Thus, in this case, staining for the β3 subunit correlates with αvβ3 expression.

Transaxial images of tumor-bearing mice 90 min after injection of [^18^F]Galacto-RGD showed a contrasting tumor on the right flank of the mouse, reflecting αvβ3-targeted tracer accumulation on endothelial cells of the tumor vasculature (Figure 2B). Moreover, we demonstrated receptor-specific tracer accumulation at the tumor site by injecting 18 mg of the pentapeptide cyclo(-Arg-Gly-Asp-DPhe-Val-) per kilogram of mouse 10 min prior to tracer injection. After blocking tracer accumulation, we found approximately 25% of the initial activity in the tumor (0.28 ± 0.05% injected dose per gram versus 1.07 ± 0.33% injected dose per gram).

### Studies in Humans

**Figure 3:**
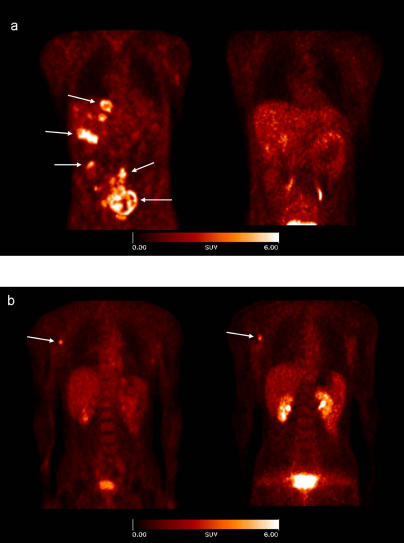
Comparison of [^18^F]FDG and [^18^F]Galacto-RGD Scans Coronal image sections, acquired 60 min p. i. (A) Patient with malignant melanoma stage IV and multiple metastases in liver, skin, and lower abdomen (arrows): marked uptake of [^18^F]FDG in the lesions (left), but no uptake of [^18^F]Galacto-RGD (right). (B) Patient with malignant melanoma stage IIIb and a solitary lymph node metastasis in the right axilla (arrow): intense uptake of both [^18^F]FDG (left) and [^18^F]Galacto-RGD (right).

For the initial evaluation in humans, we imaged nine patients (five with malignant melanomas, two with sarcomas, one with osseous metastasis from renal cell carcinoma, and one with villonodular synovitis) with approximately 185 MBq of [^18^F]Galacto-RGD. For all patients, rapid, predominantly renal excretion was observed, resulting in fast tracer elimination from blood and low tracer concentration in most of the organs. Besides the kidneys (SUV = 5.5 ± 3.7; 79 min), the highest activity concentration was found in spleen (SUV = 2.5 ± 0.5; 79 min p.i.), liver (SUV = 2.4 ± 0.5; 79 min p.i.), and intestine (SUV = 2.1 ± 0.8; 79 min p.i.). In tumor lesions, tracer accumulation showed great heterogeneity, with SUVs ranging from 1.2 to 10.0. The SUV in the villonodular synovitis was 3.2. The radioactivity was retained in the tumor tissue for more than 60 min (Table 1), whereas in all other organs a decrease of activity concentration was observed over time. Tumor/blood and tumor/muscle ratios 79 min p. i. were 3.8 ± 2.6 and 8.8 ± 6.0, respectively. Although for one melanoma patient multiple lesions were detected by the [^18^F]FDG scan, which indicates viable tumor cells, no activity accumulation was found using [^18^F]Galacto-RGD (Figure 3A). For other patients, however, similar uptake patterns were observed for [^18^F]FDG and [^18^F]Galacto-RGD (Figure 3B). The metabolite analysis of blood samples 10, 30, and 120 min p. i. showed in the soluble fractions more than 96% intact tracer (*n* = 4) over the whole observation period and confirmed our preclinical data [27]. An effective radiation dose of 18.0 ± 3.2 μSv/MBq was calculated on the basis of the patient data (*n* = 5). The highest absorbed dose was found in the urinary bladder wall (0.20 ± 0.04 mGy/MBq).

**Table 1:**
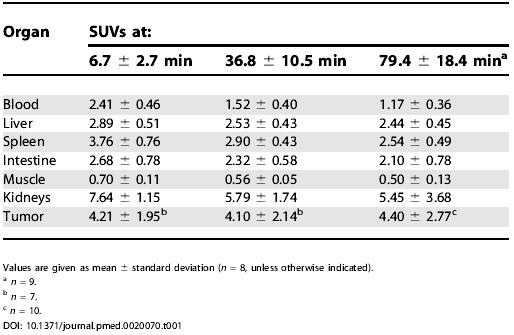
SUVs Determined Approximately 5 min, 35 min, and 75 min Post Injection

Values are given as mean ± standard deviation (*n* = 8, unless otherwise indicated)

^a^ *n* = 9

^b^ *n* = 7

^c^ *n* = 10

**Figure 4:**
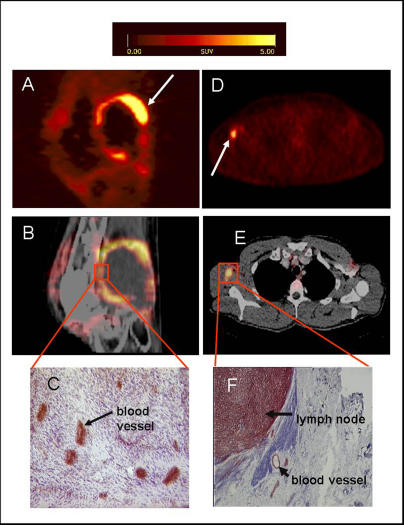
Correlation of Tracer Accumulation and αvβ3 Expression (A–C) patient with a soft tissue sarcoma dorsal of the right knee joint. (A) The sagittal section of a [^18^F]Galacto-RGD PET acquired 170 min p. i. shows circular peripheral tracer uptake in the tumor with variable intensity and a maximum SUV of 10.0 at the apical-dorsal aspect of the tumor (arrow). (B) The image fusion of the [^18^F]Galacto-RGD PET and the corresponding computed tomography scan after intravenous injection of contrast medium shows that the regions of intense tracer uptake correspond with the enhancing tumor wall, whereas the non-enhancing hypodense center of the tumor shows no tracer uptake. (C) Immunohistochemistry of a peripheral tumor section using the anti-αvβ3 monoclonal antibody LM609 demonstrates intense staining predominantly of tumor vasculature. (D–F) patient with malignant melanoma and a lymph node metastasis in the right axilla. (D) The axial section of a [^18^F]Galacto-RGD PET acquired 140 min p. i. shows intense focal uptake in the lymph node (arrow). (E) Image fusion of the [^18^F]Galacto-RGD PET and the corresponding computed tomography scan after intravenous injection of contrast medium. (F) Immunohistochemistry of the lymph node using the anti-αvβ3 monoclonal antibody LM609 demonstrates intense staining predominantly of tumor cells and also blood vessels.

Immunohistochemical staining of sections obtained from tumor tissue after surgery using an anti-αvβ3 antibody showed αvβ3 expression on the endothelial cells of the tumor vasculature (6/6), and for two patients expression on the tumor cells as well (2/6) (Figure 4). The density of αvβ3-positive vessels showed wide variation intraindividually and between individual cases. Light microscopic quantification revealed between one (inflammation of the knee due to previous operation) and 35 (soft tissue sarcoma of the knee, same patient) αvβ3-positive vessels per microscopic field. Moreover, in the six cases under analysis, density of immunohistochemically determined αvβ3-positive vessels was significantly associated with tracer accumulation as determined by SUV analysis (*r* = 0.94, *p* = 0.005).

## Discussion

Recently, we demonstrated that radiolabeled RGD peptides allow receptor-specific monitoring of αvβ3 expression in murine tumor models [24,25,26,27,35]. Here we have translated these findings to the clinical setting and for the first time, to our knowledge, demonstrated noninvasive imaging of αvβ3 expression in patients with malignant tumors. Furthermore, we have shown that the activity accumulation in the tumor correlates with the receptor density, determined by immunohistochemistry and Western blotting. This indicates that a noninvasive quantitative determination of αvβ3 expression is feasible. Furthermore, we have demonstrated in a squamous cell carcinoma model that the sensitivity of PET is adequate to image expression of αvβ3 in the tumor vasculature. This indicates that PET with [^18^F]Galacto-RGD can be applied to study αvβ3 expression during angiogenesis.

The correlation between [^18^F]Galacto-RGD uptake in the tumor and αv expression shows considerable scattering. This is probably due to several factors. As for any imaging probe, tumor uptake of [^18^F]Galacto-RGD is not only influenced by the expression of the αvβ3 integrin, but also by unspecific factors such as perfusion and vascular permeability. Furthermore, heterogeneous tracer uptake within a tumor, e.g., due to the presence of necrotic areas, will influence the correlation between [^18^F]Galacto-RGD uptake and αv expression, since separate samples were used for measurements of tracer uptake and quantitative assessment of αv expression. Finally, the present study evalutated [^18^F]Galacto-RGD uptake only at a fixed time, 90 min p. i. Imaging of the dynamics of [^18^F]Galacto-RGD accumulation in the tumor tissue and tracer kinetic modeling may allow a better quantitative assessment of αvβ3 expression by PET imaging, and this approach should be evaluated in animal models as well as in patients. Nevertheless, the significant correlation between the uptake of [^18^F]Galacto-RGD at a fixed time after injection and αvβ3 expression is very important for clinical studies, since it suggests that estimates of αvβ3 expression levels may be obtained from simple whole-body PET scans.

It has been shown that the highly bent integrin conformation is physiological and has low affinity for biological ligands, such as fibrinogen and vitronectin. Inside-out and outside-in signaling involve a switchblade-like opening to an extended structure with high affinity for endogenous ligands, as well as integrin antagonists (for overview see [36]). The inside-out activation is induced by conformational changes in the membrane-proximal regions of the α and β subunit (e.g., by intracellular proteins like talin). Outside-in signaling is triggered by Mn^2+^, which defines by quaternary rearrangements a pathway for communication from the ligand-binding site to the cytoplasmatic proximal segments. However, it is also reported that cyclo(-Arg-Gly-Asp-DPhe-Val-), in addition to binding to the high-affinity conformer, can bind to the low-affinity conformation when used at concentrations far above its dissociation constant, resulting in a similar activation as found for Mn^2+^. The nanomolar concentration used in our radiotracer approach is approximately 10,000-fold lower than that reported for the activation of the low-affinity conformation. Thus, PET with [^18^F]Galacto-RGD is expected to provide information not only about the expression of αvβ3 but also about the functional status of this integrin.

The glycopeptide [^18^F]Galacto-RGD showed high metabolic stability in patients and rapid, predominantly renal elimination, resulting in good tumor/background ratios and, thus, in high-quality images. Moreover, this finding confirms the general advantage of the glycosylation approach [24,26,37,38,39] in designing peptide-based tracers with favorable imaging properties for clinical applications. Another approach to optimize pharmacokinetics is based on the conjugation of polyethyleneglycol [40,41,42,43,44,45]. It has been demonstrated that such polyethyleneglycolated peptides also improve pharmacokinetics and tumor retention. However, a direct comparison of tracers resulting from the different strategies has not yet been carried out.

The correlation between regional tracer uptake in the lesion and density of αvβ3-positive vessels confirms that this technique allows not only visualization but also noninvasive quantitative assessment of the integrin expression. Interestingly, our study demonstrated high both inter*-* and intraindividual variances in tracer accumulation in the different lesions, indicating a great diversity in receptor expression. This finding demonstrates the value of noninvasive techniques for appropriate selection of patients entering clinical trials of αvβ3-targeting therapies. This is further emphasized by the fact that in some cases no [^18^F]Galacto-RGD uptake was found, despite viable tumor cells being detected via a [^18^F]FDG scan.

Furthermore, PET imaging with [^18^F]Galacto-RGD can be applied to assess successful blocking of αvβ3 by therapeutic agents, thereby providing essential information for the dose and dose scheduling of αvβ3 antagonists. Further studies are needed to demonstrate the impact of this new technique as a novel prognostic indicator in cancer. However, the first evidence of the prognostic value is given by Gasparini et al. [46], who found αvβ3 expression in tumor vasculature “hot spots” to be a significant prognostic factor predictive of relapse-free survival in both node-negative and node-positive patients.

αvβ3 is also found on endothelial cells during wound healing, in restenosis, in rheumatoid arthritis, and in psoriatic plaques. Thus, radiolabeled RGD peptides may be used to characterize not only malignant tumors but also inflammatory diseases. Most recently, we demonstrated in a murine model for cutaneous delayed-type hypersensitivity reaction that [^18^F]Galacto-RGD allows noninvasive assessment of αvβ3 expression in inflammatory processes [47]. Our preliminary data from a villonodular synovitis show that αvβ3 expression on endothelial cells in this lesion can be monitored in patients. Altogether, these findings indicate that [^18^F]Galacto-RGD might also become a new biomarker for disease activity in inflammatory processes.

The primary advantage of PET in imaging molecular processes is its high sensitivity combined with high penetration of the gamma radiation resulting from positron decay. Thus, PET imaging allows quantification of regional radioactivity concentrations in human studies. The optical imaging approach has an even higher sensitivity, but suffers from the low penetration of light in most tissues. This results in a very limited ability to carry out tomographic imaging and to quantify the optical signal. Thus, optical imaging is currently limited to preclinical studies in mice, whereas PET can be performed in preclinical as well as in clinical studies. Magnetic resonance imaging provides high spatial resolution and can combine morphological and functional imaging, but has approximately 1,000-fold lower sensitivity compared with PET. Thus, PET is the most appropriate technique for noninvasive determination of molecular processes in patients at the current time. Obviously, the patient is exposed to high-energy γ-rays during this procedure. However, based on our radiation dose estimates, the effective radiation dose for a [^18^F]Galacto-RGD scan is in the same range as for a [^18^F]FDG scan, an approved routine examination in the clinic in many countries [48]. In preclinical studies, different targeted magnetic resonance contrast agents have been evaluated, using either anti-αvβ3 antibody-conjugated polymerized liposomes [49] or nanoparticles [50], or nanoparticles linked with an αvβ3 peptidomimetic antagonist [51]. In those studies, depending on the contrast agent and animal model used, an average magnetic resonance signal intensity enhancement between approximately 20% and 120% was found, a finding which has not yet been confirmed in clinical studies. In our patient study using [^18^F]Galacto-RGD and PET, a 9-fold higher activity accumulation, on average, was found in the tumor than in muscle, further indicating the currently superior properties of this radiotracer for molecular imaging. Moreover, recent developments in combining PET with computed tomography or future possibilities to combine PET with magnetic resonance imaging will allow correlation of these processes with the corresponding morphology.

To further improve tumor retention of αvβ3 radioligands, multimeric RGD peptides were recently introduced. Our group developed different series of multimeric structures with up to eight RGD units linked via different spacers [40,41,42]. These multimeric RGD peptides showed increased binding affinities in vitro and improved tumor accumulation and tumor/background ratios in rodents compared with the monomeric compounds. These data and data from other groups [52,53,54] indicate that the multimeric ligand approach may be used for optimization of the performance of peptide-based tracers. However, studies in patients will be necessary to demonstrate the potential of this approach in clinical settings.

In summary, this new class of PET tracer may offer insights into molecular processes during tumor development and dissemination in preclinical as well as clinical settings, and will be a helpful tool in planning and controlling novel αvβ3-directed therapies.

## Supporting Information

Protocol S1 Approval of Ethics Committee (1.4 MB PPT).

Protocol S2 Patient Consent Form (4.2 MB PPT).

**Patient Summary**

**Background**

Tumor cells express many different molecules on their surface. These cell membrane molecules are involved in a variety of different processes, such as those that hold cells together, trigger cell death, or determine whether the tumor spreads. Some of these molecules can be tagged with radiolabeled compounds, called tracers. These tracers can show where these molecules are found and how many there are by methods such as PET and SPECT scans that don't require a biopsy, i.e., are not invasive. These methods can then be used for planning treatment with anti-cancer drugs that bind these molecules

**What Did the Investigators Do?**

They induced tumors in mice and injected them with a tracer for one cell surface molecule—an integrin. They showed that the amount of the molecule on the tumor could be measured by the intensity of tracer seen on a PET scan. They also showed that the same molecule was present on the new blood vessels that tumors produce. In a small study of patients with various tumors, including melanomas, the researchers found that the same tracer could be used to measure the expression of the integrin on tumor cells as well as on endothelial cells, such as those found in blood vessels, and hence measure the amount of new vessels in the tumors.

**What Does This Mean for Patients?**

This tracer could be useful to determine integrin expression noninvasively, to determine how many new vessels tumors have, to get information for planning anti-cancer therapies targeting integrin, and to study response to anti-cancer drugs. However, this study involved only nine patients, so much more work will need to be done before such a technique is shown to be generally reliable.

**Where Can I Get More Information?**

The National Cancer Institute has information on melanomas for patients: http://www.nci.nih.gov/cancertopics/pdq/treatment/melanoma/patient

Radiology Info explains PET scanning: http://www.radiologyinfo.org/content/petomography.htm

## Abbreviations

[^18^F]FDG: [^18^F]fluorodeoxyglucose
PET: positron emission tomography
p. i.: post injection
SUV: standardized uptake value
