# Supplementary figures and images for: Noninvasive Visualization of the Activated αvβ3 Integrin in Cancer Patients by Positron Emission Tomography and [18F]Galacto-RGD
Source: PLoS Med. 2005 Mar 29;2(3):e70. doi: 10.1371/journal.pmed.0020070 (PMC1069665; doi:10.1371/journal.pmed.0020070)

## Slide 1
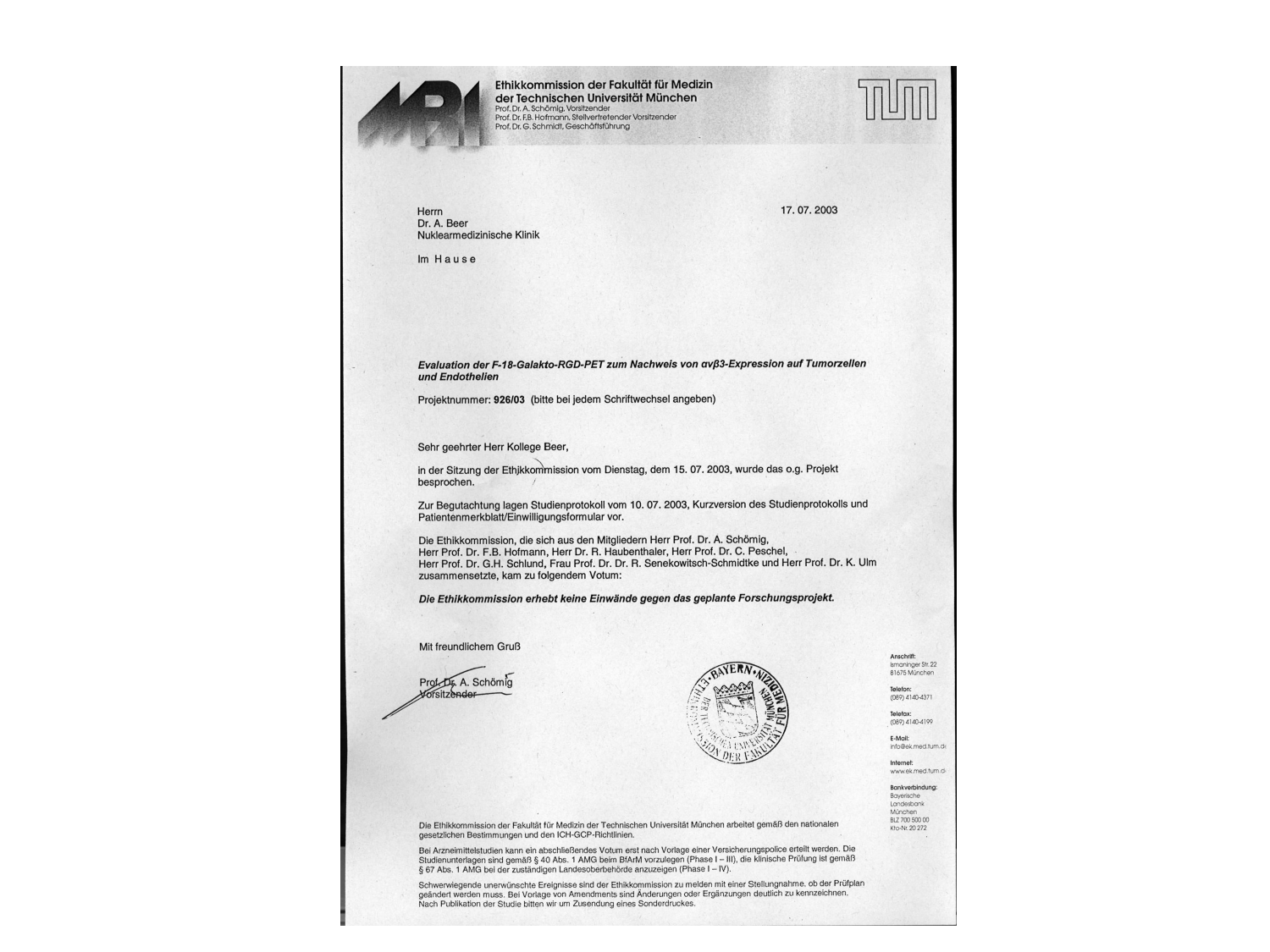

## Slide 2
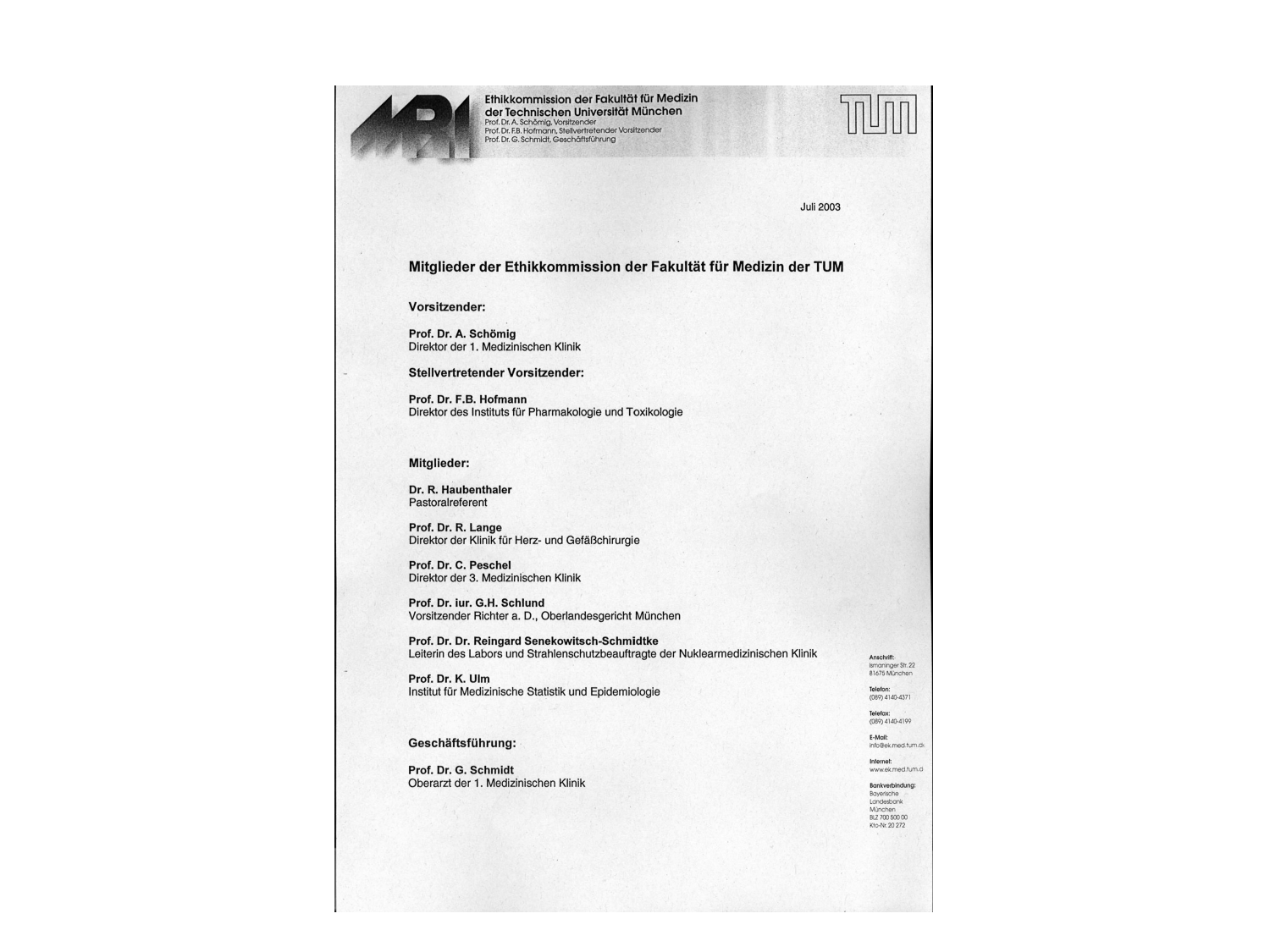

Supplement: Protocol S1 — (1.4 MB PPT). [file pmed.0020070.sd001.ppt]

## Slide 1
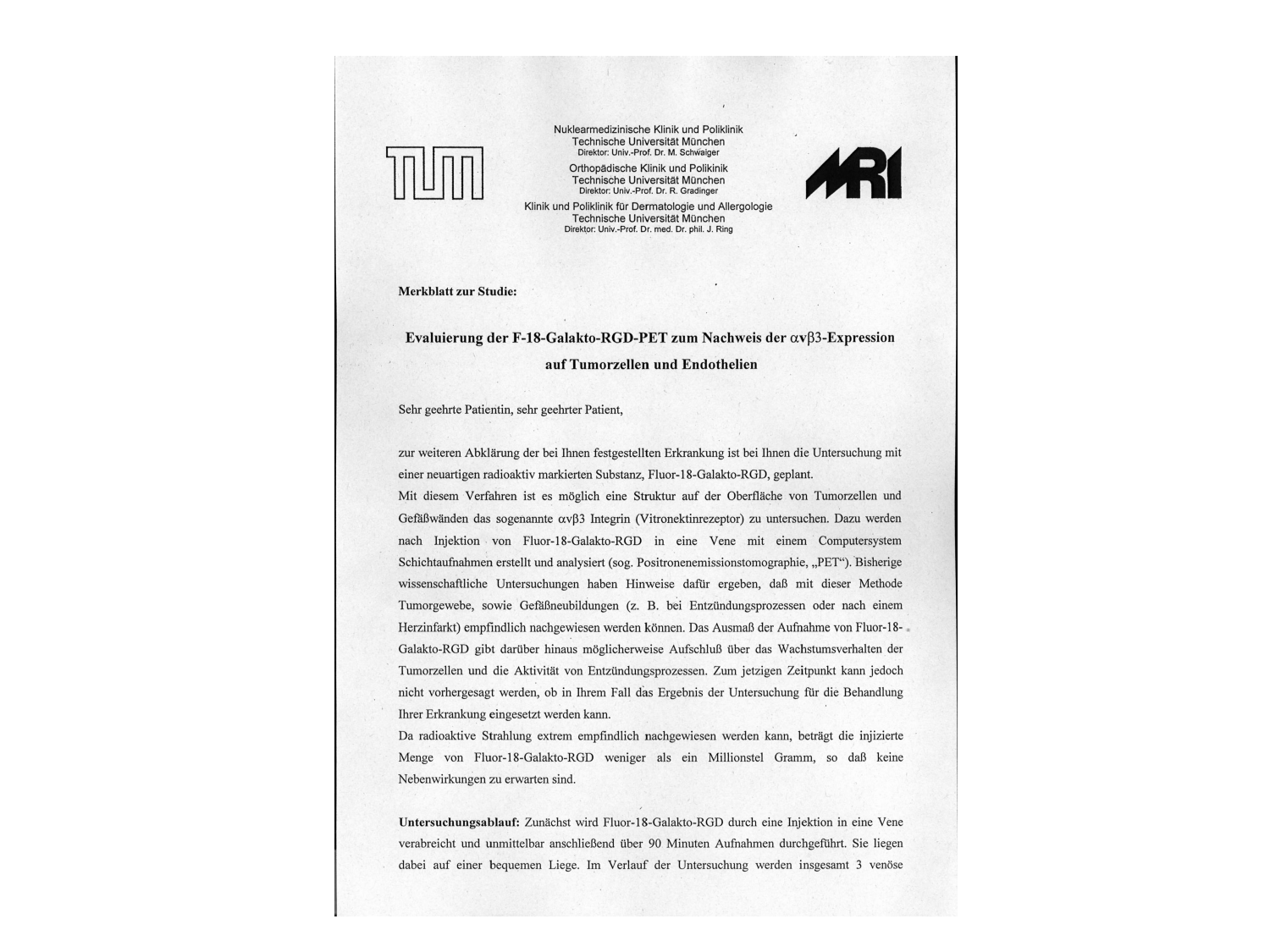

## Slide 2
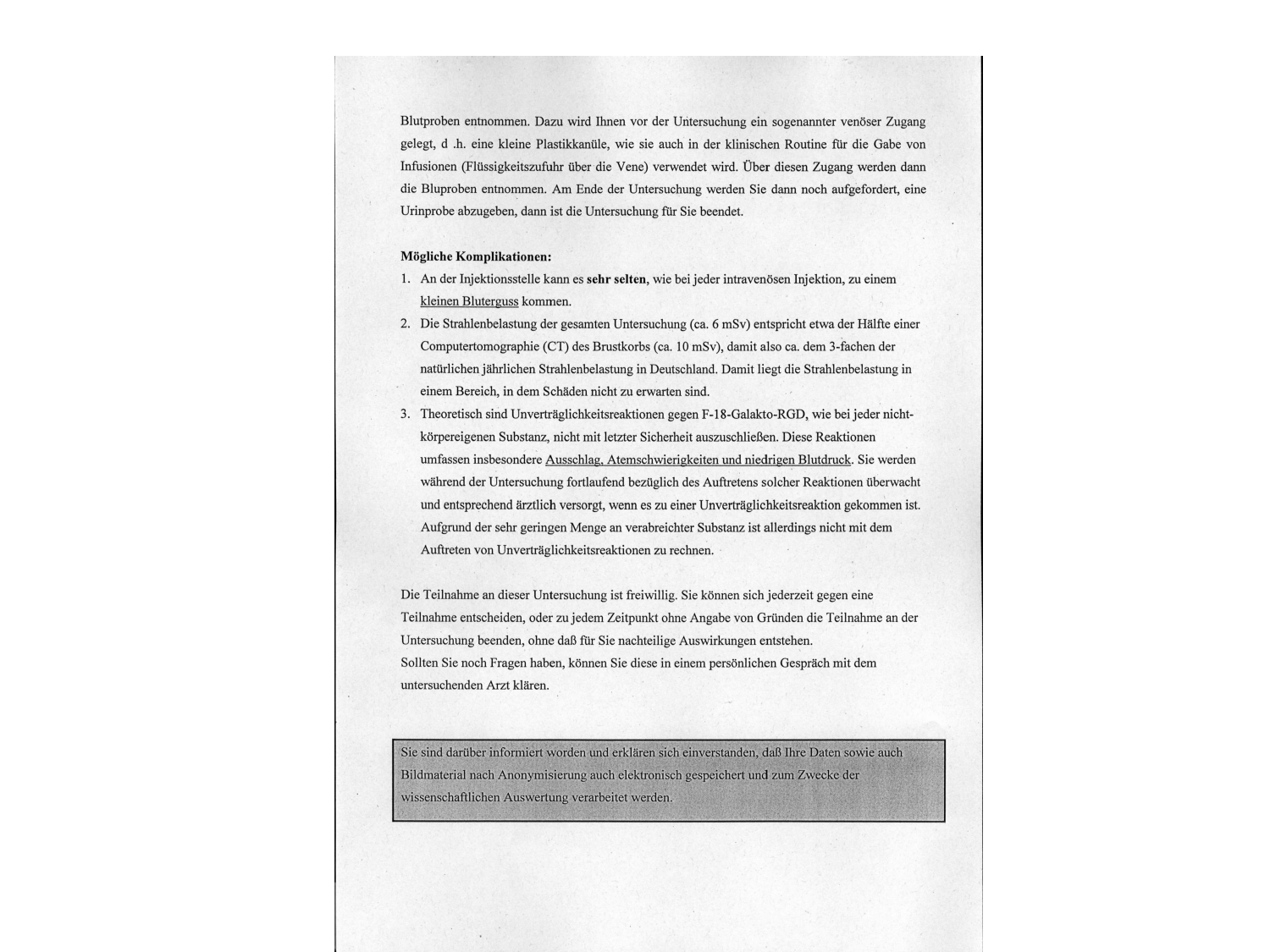

## Slide 3
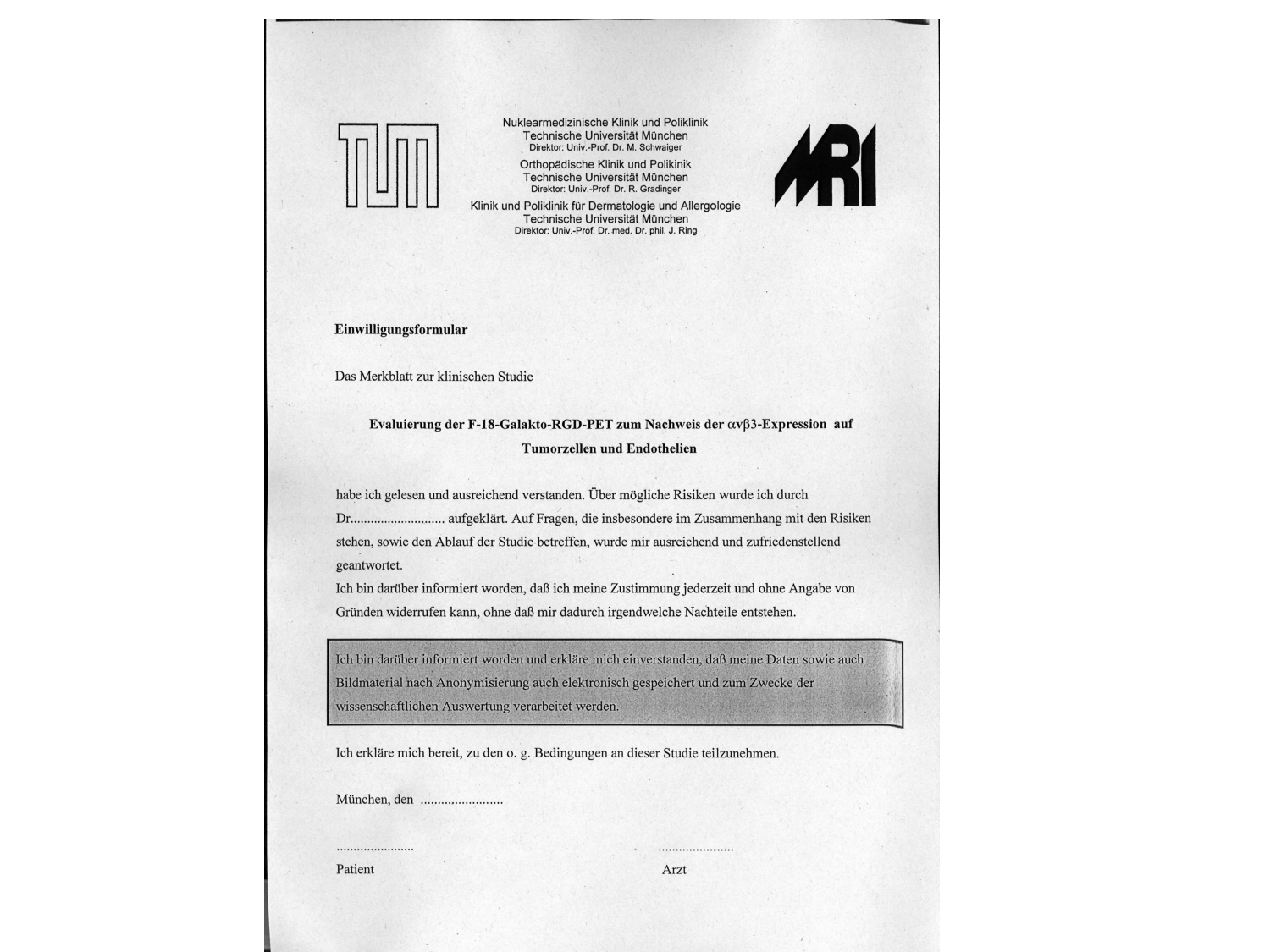

Supplement: Protocol S2 — (4.2 MB PPT). [file pmed.0020070.sd002.ppt]
